# Botanical Formulation HX109 Ameliorates TP-Induced Benign Prostate Hyperplasia in Rat Model and Inhibits Androgen Receptor Signaling by Upregulating Ca^2+^/CaMKKβ and ATF3 in LNCaP Cells

**DOI:** 10.3390/nu10121946

**Published:** 2018-12-07

**Authors:** Seonung Lim, Wonwoo Lee, Doo Suk Lee, In-Jeong Nam, Nayoung Yun, Yoonseon Jeong, Taewoong Rho, Sunyoung Kim

**Affiliations:** 1 School of Biological Sciences, Seoul National University, Seoul 151-742, Korea; kindman10@snu.ac.kr; 2 ViroMed Co. Ltd., Building 203, Seoul National University, Seoul 151-742, Korea; wwlee@viromed.co.kr (W.L.); dslee@viromed.co.kr (D.S.L.); ijnam@viromed.co.kr (I.-J.N.); nyyun@viromed.co.kr (N.Y.); ysjeong@viromed.co.kr (Y.J.);; 3 College of Pharmacy and Integrated Research Institute of Pharmaceutical Sciences, The Catholic University of Korea, Bucheon 14662, Korea; karlwho@naver.com

**Keywords:** HX109, BPH, AR signaling, CaMKKβ, ATF3

## Abstract

Benign prostatic hyperplasia (BPH) is a common disease in the elderly male population throughout the world. Among other factors, androgen dysregulation has been known to play major roles in its pathogenesis. HX109 is a botanical formulation prepared from a mixture of *Taraxacum officinale*, *Cuscuta australis*, and *Nelumbo nucifera*, which have traditionally been used—usually along with other plants—to treat urinary diseases. An ethanol extract was prepared from a mixture of these three plants, and its quality was controlled through cell-based bioassays and by quantification of several marker compounds by high-performance liquid chromatography (HPLC). In the testosterone propionate (TP)-induced prostate hyperplasia rat model, oral administration of HX109 ameliorated prostate enlargement and histological changes induced by TP. In LNCaP cells, a human prostate epithelial cell line, HX109 repressed AR-mediated cell proliferation and the induction of androgen receptor (AR) target genes at the transcriptional level without affecting the translocation or expression of AR. Such effects of HX109 on AR signaling were mediated through the control of activating transcriptional factor 3 (ATF3) expression, phosphorylation of calcium/calmodulin-dependent protein kinase kinase β (CaMKKβ), and increases in intracellular calcium, as evidenced by data from experiments involving ATF3-specific siRNA, CaMKKβ inhibitor, and calcium chelator, respectively. Taken together, our data suggest that HX109 might be used as a starting point for developing therapeutic agents for the treatment of BPH.

## 1. Introduction

Benign prostatic hyperplasia (BPH) is one of the most common chronic diseases in the aged male population throughout the world. It is reported that 50% of men over the age of 50 have enlarged prostates, with the incidence increasing with age, and reaching 90% in men over 90 [1,2]. BPH is characterized by a histological change in the prostate architecture and variable growth of the prostate size. Increase in prostate size tightens the urethra and induces lower urinary tract symptoms such as nocturia, dysuria, and bladder obstruction [3].

Although the worldwide prevalence of BPH is high, the etiology of BPH has yet to be fully elucidated. The progression of BPH involves various factors including aging, hormonal changes, metabolic syndrome, oxidative stress, and inflammation [4,5,6]. Among these, androgen dysregulation is particularly important, as it can induce prostate cell proliferation. In the prostate, testosterone is converted to dihydrotestosterone (DHT) by 5α-reductase. Both DHT and testosterone bind to androgen receptor (AR) and promote protein synthesis involved in the cellular growth of prostate cells [7]. Since androgen/AR signaling plays an important role in the pathogenesis of BPH, many therapeutic agents are being developed to target this pathway.

AR is a receptor of androgen normally present in the cytoplasm that translocates into the nucleus upon binding the androgen. In the nucleus, AR regulates gene expression by binding to androgen receptor response elements (AREs) located in the promoter and enhancer region of various target genes [8]. Androgen-mediated gene expression is affected by a number of different factors. Activating transcriptional factor 3 (ATF3), known as a common stress response mediator, is a member of the ATF/cAMP response element-binding protein (CREB) family of transcriptional factor [9]. ATF3 represses AR transactivation by binding to the transcription domain of the AR. It was reported that the loss of ATF3 results in increased prostate cell proliferation as well as the transcription of androgen-target genes [10]. Calcium/calmodulin-dependent protein kinase kinase β (CaMKKβ), which is activated by Ca^2+^/calmodulin, is also known to influence AR signaling. It has been reported that CaMKKβ overexpression inhibits AR-mediated gene expression, whereas the knockdown of CaMKKβ enhances AR-signaling and proliferation [11].

The most frequently prescribed drugs for BPH are alpha blockers and 5α-reductase inhibitors (5ARIs). Alpha blockers are used in initial therapy for improving urine flow, but they do not reduce prostate size [12]. 5ARIs, represented by finasteride, block prostate enlargement by blocking the conversion of testosterone to DHT. 5ARIs are more effective and have a longer lasting effect than alpha-blockers, but have been reported to produce side effects such as the loss of libido and ejaculation, and impotence [13,14]. As such, there is a strong need for the development of safer and more efficacious agents for BPH.

Because BPH is a chronic disease caused by multiple factors, plants may provide an interesting source of developing therapeutic agents. Because botanical medicines contain multiple components, they can potentially interact with multiple cellular targets. Since many plants have traditionally been used as human medicines, historical evidence of their safety and efficacy is also readily available [15]. HX109 is an ethanol extract prepared from three plants: *Taraxacum officinale*, *Cuscuta australis*, and *Nelumbo nucifera*. These plants were selected on the basis of previous publications describing their activities and functions, and information available from traditional phytomedical practices in Asia. *Taraxacum officinale* has been widely used to treat urinary and renal diseases because of its diuretic, choleretic, anti-inflammatory, and anti-carcinogenic effects [16]. It has also been reported to inhibit prostate cancer cell proliferation and counteract inflammation [17,18]. *Cuscuta australis* has been used as a tonic to treat urinary complaints, such as frequent urination and involuntary ejaculation [19]. The aqueous extract of *Nelumbo nucifera* has been reported to have antioxidant and anti-steroid properties that may inhibit androgen signaling [20,21,22]. Therefore, we hypothesized that the combination of these three plants might be effective in ameliorating BPH symptoms.

In this study, we investigated the therapeutic potential of HX109 in a TP-induced BPH rat model by measuring prostate weight and the protein level of prostate specific antigens (PSAs). After observing significant improvements in BPH condition in rat model, LNCaP cells, a human prostate epithelial cell line, were used to understand the underlying mechanism. 

## 2. Materials and Methods 

### 2.1. Preparation of HX109

All plants used in the preparation of HX109 were purchased from Humanherb (Gyeongsan, Korea) and authenticated through the plant identification services of Plant DNA Bank in Korea (PDBK, Seoul, Korea) using their genome sequences. HX109 was prepared by mixing *Taraxacum officinale*, *Cuscuta australis*, and *Nelumbo nucifera* at a ratio of 2:1:1. The combination of plants (total dry weight, 60 g) was extracted with 600 mL of 25% EtOH at 20 °C for 8 h. The extract was filtered with 10-μm cartridge paper and concentrated using a rotary evaporator (Eyela, Tokyo, Japan), followed by a freeze-drying process. This process generally produced approximately 8.5 g of brown powder with a yield of about 14%. The voucher specimens used in this study were deposited in the herbarium of ViroMed Co., Ltd. (Seoul, Korea).

### 2.2. High-Performance Liquid Chromatography (HPLC) Analysis

High-performance liquid chromatography analysis was employed to validate the quality of HX109. Reference standards for chicoric acid, maltol, dihydrophaseic acid, and isoschaftoside were used for qualitative and quantitative analyses of HX109. Analytical samples of HX109 were studied by HPLC-PDA (Waters, Millford, MA, USA) with Capcell PAK C18 MG column (4.6 mm × 250 mm, 5 µm, Shiseido, Japan). Water (0.05% trifluoroacetic acid) for solvent A and acetonitrile (0.01% trifluoroacetic acid) for solvent B was used for the mobile phase. The mobile phase gradient was 5–27% B (0–10 min), 27–35% B (10–25 min), 35–100% B (25–30 min); the flow rate was 1.0 mL/min, and the injection volume was 5 µL at the concentration of 20 mg/mL. The samples were analyzed at a wavelength of 280 nm and the optimum temperature for HPLC separation was 25 °C.

### 2.3. Animals 

Ten-week old male Sprague Dawley (SD) rats weighing 330 ± 20 g were obtained from Orient Bio (Seongnam, Korea) for animal studies. Animals were housed under controlled environmental conditions: constant temperature (25 ± 2 °C), humidity (60 ± 10%), and a 12 h light/ dark cycle. All experiments were performed according to the guidelines set by the International Animal Care and Use Committee at Seoul National University (Approval Number: SNU-131111-5-1). 

### 2.4. TP-Induced Benign Prostate Hyperplasia Rat Model

Rats were acclimatized for 1 week, followed by bilateral orchiectomies to prevent the influence of endogenous testosterone. After 1 week, rats were divided into five groups: NC, BPH, HX200, HX300, and Fina (*n* = 5 per group). Prostatic hyperplasia was induced in four groups (BPH, HX200, HX300, Fina) by subcutaneous injection of 3 mg/kg of testosterone propionate (TP) (Tokyo Chemical Industry, Tokyo, Japan) dissolved in cottonseed oil (Sigma-Aldrich, St. Louis, MO, USA) every three days. The NC group received only cottonseed oil in order to provide similar subcutaneous injection conditions in all groups. During the induction of prostate hyperplasia, rats orally received respective reagents on a daily basis for 4 weeks. The HX200 group and HX300 group were orally administrated 200 mg/kg of HX109 or 300 mg/kg of HX109. The Fina group was orally administrated 5 mg/kg of finasteride as a positive control. The NC group and BPH group were orally administrated distilled water as a vehicle. Body weight was measured once a week during the experiment. After 4 weeks, rats were sacrificed, and prostates were immediately removed and weighed.

### 2.5. H&E Staining

Prostates were fixed in 10% normalized buffered formalin (Sigma-Aldrich, St. Louis, MO, USA) and embedded in the paraffin block. Then, 6-μm paraffin sections of the prostate were stained with Hematoxylin&Eosin to analyze acinar areas. The size of each acinus was measured by ImageJ software version 1.50i (National Institutes of Health, Bethesda, MD, USA). 

### 2.6. Enzyme-Linked Immunosorbent Assay (ELISA)

To measure DHT and PSA levels, ELISA kits specific to DHT (ALPCO Diagnostics, Salem, NH, USA) and PSA (Cusabio, Houston, TX, USA) were used according to the manufacturer’s instructions. When in vivo samples were prepared, sera were used to detect DHT, and levels were expressed as pg/mL. Prostate samples were homogenized using T-PER tissue protein lysis buffer (Thermo Fisher Scientific, Woburn, MA, USA) containing a protease inhibitor (Roche, Basel, Switzerland) and a phosphatase inhibitor (Roche, Basel, Switzerland). After preparation, samples were centrifuged at 12,000 rpm for 10 min at 4 °C and the supernatants were used to detect DHT and PSAs. Values from prostate proteins were normalized by total proteins and expressed as pg/mg protein. 

### 2.7. Cell Culture and Reagents

LNCaP human prostate cancer cell lines were purchased from American Type Culture Collection (ATCC, Manassas, VA, USA). Cells were cultured in RPMI 1640 medium supplemented with a 10% heat-inactivated fetal bovine serum, HEPES (10 mM), penicillin, and streptomycin in a humidified 5% CO_2_ atmosphere at 37 °C. To examine the effects of TP, cells were cultured in phenol red-free RPMI 1640 containing 5% charcoal stripped serum (CSS) (TCB, Long Beach, CA, USA) for 24 h, and 100 nM TP was then added to the medium. STO-609 (a CaMKKβ inhibitor, Tocris Bioscience, Ellisville, MO, USA) was used at 30 μM and BAPTA-AM (a calcium chelator, Sigma-Aldrich, St. Louis, MO, USA) was used at 20 μM for the experiment. 

### 2.8. RNA Isolation and qRT-PCR

Total RNAs were prepared from LNCaP cells using Trizol reagent (Invitrogen, Carlsbad, CA, USA) according to the manufacturer’s protocol. One microgram of RNA was converted to cDNA using oligo dT primers (QIAGEN, Hilden, Germany) and Reverse Transcriptase XL (avian myeloblastosis virus (AMV)) (Takara, Kusatsu, Japan). Real-time quantitative RT-PCR was performed with SYBR Premix (Takara, Kusatsu, Japan) and Thermal Cycler Dice Real Time System TP800 (Takara, Kusatsu, Japan). PCR conditions were denaturation at 95 °C for 5 s, followed by annealing and extension at 60 °C for 30 s. The sequences of synthesized PCR primer sets (Bioneer Co. Ltd., Seoul, Korea) for KLK3 (hPSA) were 5′-GTGTGTGGACCTCCATGTTATT-3′ and 5′-CCACTCACCTTTCCCCTCAAG-3′; for ATF3 were 5′-AAGAACGAGAAGCAGCATTTGA-3′ and 5′-TTCTGAGCCCGGACAATACAC-3′; for KLK2 were 5′-ATGTGTGCTAGAGCTTACTC-3′ and 5′-AAGTGGACCCCCAGAATCAC-3′; for TMPRSS2 were 5′-GGACAGTGTGCACCTCAAAGAC-3′ and 5′-TCCCACGAGGAAGGTCCC-3′; for DHCR24 were 5′-GAGGCAGCTGGAGAAGTTTG-3′ and 5′-CTTGTGGTACAAGGAGCCATC-3′; for NKX3.1 were 5′-CCTCCCTGGTCTCCGTGTA-3′ and 5′-TGTCACCTGAGCTGGCATTAC-3′.

### 2.9. Western Blot

After treatment with TP and HX109, LNCaP cells were washed with cold PBS lysed with radioimmunoprecitation(RIPA) lysis buffer (Sigma-Aldrich, St. Louis, MO, USA) containing a protease inhibitor (Roche, Basel, Switzerland) and a phosphatase inhibitor (Roche, Basel, Switzerland). Equal amounts of protein were then separated by 10% SDS-polyacrylamide gel and electrophoretically transferred to polyvinylidene difluoride (PVDF) membranes (Millipore, Burlington, MA, USA). The membranes were blocked with 5% bovine serum albumin (Gibco, Waltham, MA, USA) in TBST buffer (1 M Tris-HCl (pH 7.4), 0.9% NaCl, and 0.1% Tween 20) for 1 h and incubated with primary antibodies diluted in a 3% BSA blocking solution overnight at 4 °C. Membranes were then treated with HRP-conjugated anti-mouse or anti-rabbit IgG (1:100,000; Sigma-Aldrich, St. Louis, MO, USA) for 1 h, and protein bands were visualized with an enhanced chemiluminescence solution (Millipore, Burlington, MA, USA) and X-Omat film (Kodak, Rochester, NY, USA).

### 2.10. Luciferase Reporter Plasmid Assay

An inducible AREs-responsive luciferase reporter assay kit was purchased from QIAGEN (Valencia, CA, USA), and the assay was performed as described previously [14]. LNCaP cells were briefly transfected with ARE-reporter plasmid or negative control plasmid using lipofectamine 3000 (Invitrogen) according to the manufacturer’s protocol. Twenty-four hours after transfection, the cells were treated with TP (100 nM) and various concentrations of HX109 for 18 h. Cell lysates were prepared, and a luciferase activity assay was performed using the dual luciferase reporter assay system (Promega, Madison, WI, USA) and microplate luminometer (MicroLumat Plus LB96V, Berthold, Germany) according to the manufacturer’s protocol. The data are shown as the ratio of firefly luciferase activity to Renilla luciferase activity (Fluc/Rluc).

### 2.11. Extraction of Nuclear and Cytoplasmic Fractions 

Fractionation and extraction of nuclear and cytoplasmic proteins from LNCaP cells treated with TP and HX109 for 3 h were performed using NE-PER Nuclear and Cytoplasmic Extraction Reagents (Thermo Fisher Scientific, Woburn, MA, USA) according to the manufacturer’s protocol. 

### 2.12. siRNA Transfection

The siRNA specific to ATF3 and scrambled siRNA (Thermo Fisher Scientific, Woburn, MA, USA) were transfected into LNCaP cells using RNAiMAX (Thermo Fisher Scientific, Woburn, MA, USA) according to the manufacturer’s instructions. Twenty-four hours after the siRNA mediated knockdown of ATF3, cells were briefly treated with TP and HX109 and then subjected to further analysis. Knockdown efficiency was evaluated using an antibody against ATF3 (1:1000, Cell Signaling Technology, Danvers, MA, USA).

### 2.13. Calcium Assay

For the calcium assay, LNCaP cells were plated at 5 × 10^4^ cells per well in a 24-well CellBIND plate containing phenol red-free RPMI with 10% fetal bovine serum (FBS). Twenty-four hours later, cells were treated with TP and 1 mg/mL HX109. After 1 and 5 min, cells were washed with PBS and lysed with PBS containing 0.5% Triton X. The calcium levels of the cell lysates were measured using a calcium assay kit (Sigma-Aldrich, St. Louis, MO, USA) according to the manufacturer’s protocol.

### 2.14. Statistical Analysis

All values are presented as mean ± S.E.M. from three independent experiments. Statistical significance was determined using unpaired Student’s *t*-test or one-way ANOVA with Turkey correction, provided by the GraphPad Prism software version 7 (GraphPad, San Diego, CA, USA). Data were considered statistically significant if the *p*-value was <0.05.

## 3. Results

### 3.1. Quality of HX109 Is Monitored by HPLC Analysis and Cell-Based Bioassay

**Figure 1:**
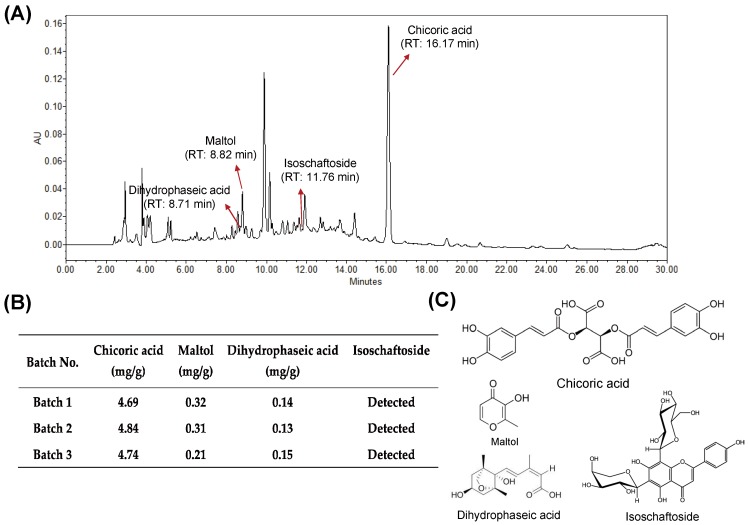
Preparation of HX109 was standardized using marker compounds. HX109 was dissolved in 50% methanol at a concentration of 20 mg/mL and subjected to HPLC analysis. (**A**) Representative HPLC chromatogram of HX109. HPLC was performed as described in the Materials and Methods section. The marker compounds in HX109 were identified by comparison with the reference standard and the peaks of marker compounds are indicated by arrows. RT, retention time. (**B**) Quantification of marker compounds in HX109. (**C**) Molecular structure of marker compounds.

To establish batch-to-batch consistency of research grade HX109, one representative marker compound from each plant (chicoric acid for *Taraxacum officinale*, maltol for *Cuscuta australis*, and dihydrophaseic acid for *Nelumbo nucifera*) was used (Figure 1C), based on previously published information [23,24,25]. In addition, isoschaftoside was used as a qualitative marker of *Nelumbo nucifera* [26]. The marker compounds were analyzed using HPLC (Figure 1A), and only the extracts containing these compounds within the set range (4.8 ± 0.2 mg/g for chicoric acid, 0.3 ± 0.1 mg/g for maltol, and 0.1 ± 0.05 mg/g for dihydrophaseic acid) were used for this study (Figure 1B). The identifications of these marker compounds were further confirmed by mass spectrometer (see Figures S1 and S2 in the Supplementary Materials). 

In addition, the quality of HX109 was biologically controlled using cell-based bioassays. LNCaP cells were treated with TP in the presence of various concentrations of HX109 and the effect on PSA secretions was determined to calculate the half maximal inhibitory concentration(IC_50_) value (see Figure S3 in the Supplementary Materials). Only HX109 preparations showing IC_50_ values between 2.2 to 2.4 mg/mL were used for the experiments.

### 3.2. HX109 Ameliorates TP-Induced Benign Prostatic Hyperplasia in Castrated Sprague Dawley Rats 

To test the effects of HX109 on BPH, a testosterone propionate (TP)-induced BPH rat model was used. Injection of TP every three days into castrated Sprague Dawley rats for 4 weeks (designated as the BPH group) caused a significant (*p* < 0.001) increase in prostate weight and index compared to the castration-only group (NC) (Table 1). In rats orally administrated with HX109 for 4 weeks, however, both prostate weight and index decreased in a dose-dependent manner. Prostate weight was reduced by 33% in rats receiving 300 mg/kg HX109 compared to the BPH control group, while finasteride, used as a positive control, decreased it by 43%. Body weight was not affected in all groups (Table 1). 

**Figure 2:**
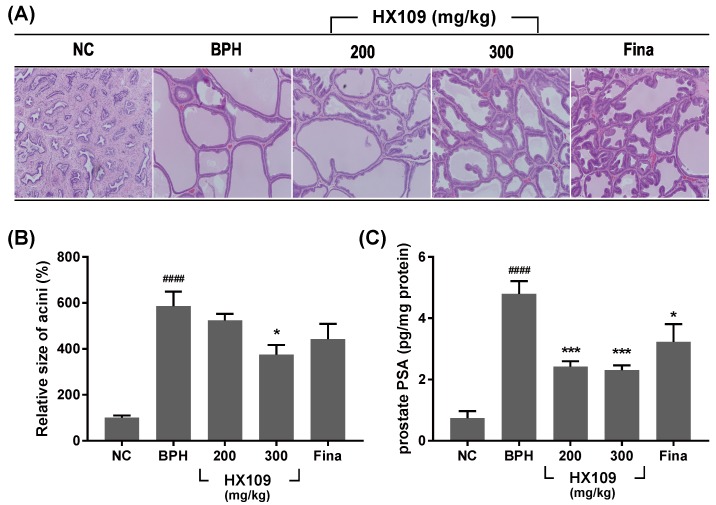
HX109 repressed TP-induced enlargement of prostate and PSA expression in rat prostate. Castrated Sprague Dawley rats were injected with 3 mg/kg every three days and orally administrated with TDW (BPH) or 200 mg/kg of HX109 or 300 mg/kg of HX109 or 5 mg/kg of finasteride (Fina). Rats injected with vehicle were used as a negative control (NC) group. (**A**) Effects of HX109 on histological changes in prostate. After measuring the final prostate weight, rats were sacrificed and prostate tissues were fixed, sectioned, and stained with hematoxylin and eosin (H&E). (**B**) Relative size of acinal area. Values were calculated from three randomly captured pictures. ^####^ *p* < 0.0001 (one-way ANOVA) compared with the NC group, * *p* < 0.05 (one-way ANOVA) compared with the BPH group. All data are shown as mean ± S.E.M. (**C**) Effects of HX109 on prostate PSA levels. PSA levels of rat prostate were measured by ELISA. Values were normalized to total proteins. *n* = 5 per group. ^####^ *p* < 0.0001 (one-way ANOVA) compared with the NC group, * *p* < 0.05, *** *p* < 0.001 (one-way ANOVA) compared with BPH group. All data are shown as mean ± S.E.M.

H&E staining was performed to investigate the effect of HX109 on histological changes in the prostate. As shown in Figure 2A, administration of TP increased the area of prostatic acinar 6-fold compared to the NC group, but the area size decreased by 35% following HX109 treatment (Figure 2B). The effect of HX109 on the protein levels of prostate specific antigens (PSAs), a frequently used marker of prostate enlargement, was also investigated. The PSA levels in the prostate were markedly higher in the BPH group than in the NC group. However, they were reduced in the HX109-treated group compared to the BPH group (Figure 2C). 

In the prostate, the major prostatic androgen is DHT, which is converted by 5α-reductase from testosterone [27]. When DHT levels in the serum and prostate were measured, they were increased in the BPH group compared to the NC group, and HX109 treatment had little effect on DHT levels (see Figure S4A,B in the Supplementary Materials). These results indicated that 5α-reductase might not be involved in the HX109-mediated amelioration of prostate enlargement. 

### 3.3. HX109 Suppresses Androgen-Dependent Proliferation of LNCaP Cells 

**Figure 3:**
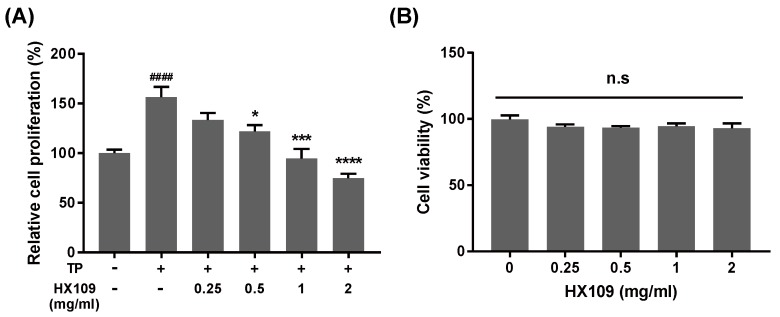
HX109 suppressed androgen-dependent proliferation of LNCaP cells. LNCaP cells were plated in culture media for 24 h followed by androgen starvation using phenol red free RPMI 1640 containing 10% charcoal-stripped serum for 24 h. After androgen starvation, LNCaP cells were treated with or without 100 nM TP and cultured in the presence of various concentrations of HX109 for 72 h. Cell proliferation was measured by WST-1 assay. (**A**) Effects of HX109 on the TP-induced proliferation of LNCaP cells. (**B**) Cytotoxicity effects of HX109. ^####^ *p* < 0.0001 (one-way ANOVA) compared with control, * *p* < 0.05, *** *p* < 0.001, **** *p* < 0.0001 (one-way ANOVA) compared with TP only. n.s, not significant. Values are normalized to control. All data are shown as mean ± S.E.M. of three independent experiments. ‘+’ means ‘treated with TP or HX109’, if it is on the right side of TP, it means ‘treated with TP’; if it is on the right side of HX109, it means ’treated with HX109’. ‘−’ means ‘not treated with TP or HX109’.

In the BPH rat model, the increase in prostate weight by TP is well known to be induced by prostate cell proliferation. Therefore, it was tested whether HX109 could control androgen-induced prostate cell proliferation. LNCaP cells were treated with various concentrations of HX109, with or without the addition of TP, and effects on cell proliferation were measured by WST-1 assay. As shown in Figure 3A, treatment with TP increased proliferation of LNCaP cells by about 56% compared to the vehicle group, but HX109 treatment inhibited TP-induced cell proliferation in a dose-dependent manner. At 2 mg/mL of HX109, TP-induced cell proliferation was inhibited by almost 50%. These effects were not due to cytotoxicity, as cell viability was not affected by HX109 (Figure 3B). These data suggested that HX109 might inhibit the androgen-dependent proliferation of LNCaP cells.

### 3.4. HX109 Inhibits Androgen-Induced PSA Expression 

**Figure 4:**
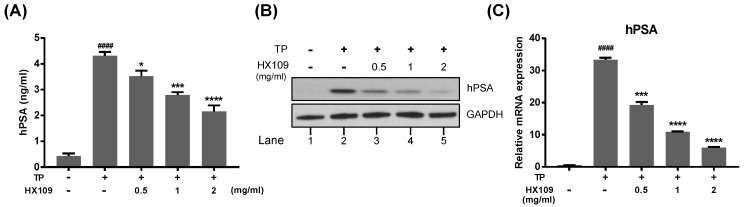
HX109 inhibited the androgen-induced PSA (KLK3) expression in LNCaP cells. LNCaP cells were treated as described in Figure 3. (**A**) Effects of HX109 on hPSA protein secretion were analyzed by ELISA after 24 h. ^####^ *p* < 0.0001 (one-way ANOVA) compared with control, * *p* < 0.05, *** *p* < 0.001, **** *p* < 0.0001 (one-way ANOVA) compared with TP only. (**B**) Effects of HX109 on hPSA protein expression. After 48 h, protein levels in cell lysate were analyzed by western blot. (**C**) Effects of HX109 on hPSA mRNA expression. The RNA levels were analyzed by quantitative RT-PCR after 24 h. Values were normalized to GAPDH for both protein and RNA analysis. ^####^ *p* < 0.0001 compared with control, *** *p* < 0.001, **** *p* < 0.0001 (one-way ANOVA) compared with TP only. All data are represented as mean ± S.E.M. of three independent experiments. ‘+’ means ‘treated with TP or HX109’, if it is on the right side of TP, it means ‘treated with TP’; if it is on the right side of HX109, it means ’treated with HX109’. ‘−’ means ‘not treated with TP or HX109’.

Androgen-dependent proliferation is regulated by androgen/androgen receptor (AR) signaling. Since PSA is the main target of AR signaling, the effect of HX109 on this protein was investigated. In the absence of TP, LNCaP produced a small amount of PSA, but treatment with 100 nM TP elevated its level by 10-fold. When treated with HX109, PSA levels in the cell culture supernatant were decreased in a dose-dependent manner, maximally by 50% at 2 mg/mL (Figure 4A). Suppression of PSA expression by HX109 was also observed by western blot (Figure 4B).

To determine whether HX109 affects the production of PSAs at a transcriptional level, the RNA level of PSAs was measured by quantitative RT-PCR. TP-induced RNA expression of PSA was reduced by HX109 in a dose-dependent manner (Figure 4C). At 2 mg/mL HX109, the RNA level of PSAs was lowered by 80% compared to TP only. To further verify the effects of HX109 on AR signaling, the RNA levels of other downstream target genes of AR—such as KLK2, TMPRSS2, DHCR24, and NKX3.1—were also measured. The expression of all target genes was highly downregulated when treated with HX109 (see Figure S5 in the Supplementary Materials). These results indicated that HX109 could effectively inhibit AR-mediated gene expression. 

### 3.5. HX109 Inhibits AR Transcriptional Activity Without Affecting AR Expression and Translocation

**Figure 5:**
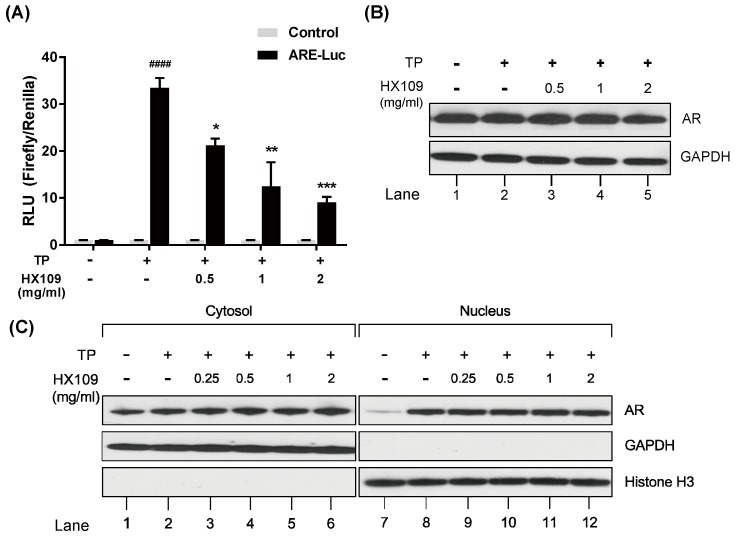
HX109 inhibited androgen receptor (AR) transcriptional activity without affecting AR expression and translocation. (**A**) Effects of HX109 on luciferase activity. LNCaP cells were transfected with control or luciferase reporter plasmid containing androgen receptor response element (ARE) sequence for 24 h. Transfected cells were treated with or without 100 nM TP in the presence of various concentrations of HX109 for 18 h. Luciferase activity was measured as described in the Materials and Methods section. ^####^ *p* < 0.0001 (one-way ANOVA) compared with control, * *p* < 0.05, ** *p* < 0.001, *** *p* < 0.0001 (one-way ANOVA) compared with TP only. (**B**) The protein levels of AR were analyzed by western blot after 24 h treatment with HX109. (**C**) Effects of HX109 on AR translocation. LNCaP cells were treated with 100 nM TP in the presence of various concentrations of HX109 for 3 h. After treatment, nuclear and cytoplasmic extracts were collected and subjected to western blot. For western blot, three independent experiments were performed, and one representative result is presented here. All values are shown as mean ± S.E.M. of three independent experiments. ‘+’ means ‘treated with TP or HX109’, if it is on the right side of TP, it means ‘treated with TP’; if it is on the right side of HX109, it means ’treated with HX109’. ‘−’ means ‘not treated with TP or HX109’.

The induction of PSAs by androgen is mediated by the binding of AR to ARE present in the PSA promoter [28]. Therefore, AR-dependent transactivation was assessed by a reporter plasmid assay in which luciferase expression is dependent on the binding of AR to ARE element. LNCaP cells were transfected with luciferase reporter construct containing three copies of ARE, and then treated with TP in the presence of HX109. As shown in Figure 5A, the level of luciferase activity was increased by TP, but was reduced by HX109 treatment in a dose-dependent manner. 

It was also tested whether HX109 inhibited the expression of AR itself. No significant change in AR expression levels was observed in cells treated with HX109 for 24 h (Figure 5B). Lastly, the effects of HX109 on AR nuclear translocation were studied by isolating cytoplasmic and nuclear proteins. In the absence of TP, AR was only presented in the cytoplasmic compartment, but translocated to the nucleus upon TP treatment. There was little difference in the nuclear levels of AR between the TP-only and HX109 groups (Figure 5C). Taken together, these results suggest that HX109 might inhibit the transcriptional activity of AR without affecting AR expression or translocation. 

### 3.6. HX109 Regulates AR Transactivation Through Upregulation of CaMKKβ and ATF3

**Figure 6:**
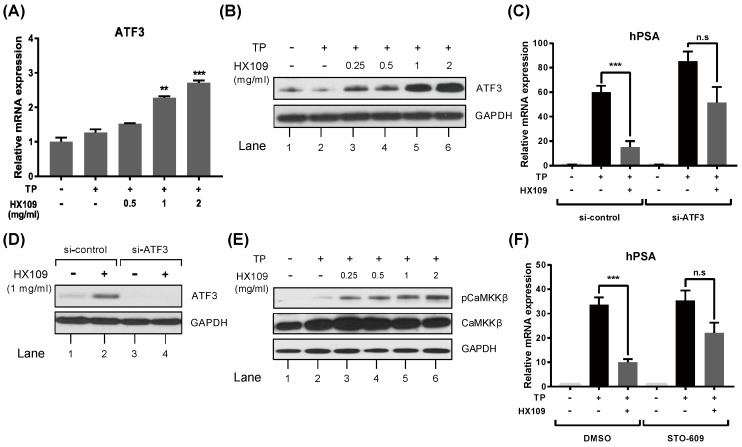
HX109 regulated AR transcriptional activity by phosphorylation of CAMKKβ and upregulation of ATF3. LNCaP cells were treated as described in Figure 3, and total RNAs and proteins were prepared and analyzed for ATF3 by quantitative RT-PCR and western blot, respectively. Effects of HX109 on ATF3 RNA (**A**) and protein (**B**) levels were measured after a 6 h or 8 h treatment with HX109, respectively. ** *p* < 0.01, *** *p* < 0.001 (one-way ANOVA) compared with TP only. LNCaP cells were transfected with ATF3 siRNA or control siRNA for 24 h and then treated with 100 nM TP in the presence of HX109 1 mg/mL. (**C**) The expression of hPSA RNA levels were measured by quantitative RT-PCR after 24 h treatment. *** *p* < 0.001. (**D**) The protein levels of ATF3 were determined by western blot after 8 h treatment. (**E**) Effects of HX109 on the phosphorylation of CAMKKβ were measured by western blot. Total proteins were prepared after 30 min of TP and HX109 treatment. (**F**) LNCaP cells were incubated with 30 μM STO-609 for 30 min and treated with 100 nM TP in the presence of 1 mg/mL HX109 for 24 h. The RNA levels of hPSA were measured by quantitative RT-PCR. Values of qRT-PCR were normalized to GAPDH. *** *p* < 0.001 (one-way ANOVA) compared with TP only. n.s, not significant. For western blot, three independent experiments were performed, and one representative result is shown here. All values are shown as mean ± S.E.M. of three independent experiments. ‘+’ means ‘treated with TP or HX109’, if it is on the right side of TP, it means ‘treated with TP’; if it is on the right side of HX109, it means ’treated with HX109’. ‘−’ means ‘not treated with TP or HX109’.

It was previously reported that CaMKKβ and ATF3 repress the transactivation of AR [10,11], and that the expression of the ATF3 gene is downregulated in human BPH tissue [29]. We first assessed the effect of HX109 on ATF3 expression. In LNCaP cells, TP had no effect on ATF3 expression, but treatment with HX109 increased the RNA (Figure 6A) and protein (Figure 6B) levels of ATF3 in a dose-dependent manner. To study the role of ATF3 in the HX109-mediated suppression of PSAs, ATF3 expression was inhibited using siRNA specific to ATF3. The knockdown efficiency of ATF3 was confirmed by western blot analysis (Figure 6C). Consistent with previous reports showing the repression of AR transactivation by ATF3, the inhibition of ATF3 expression increased TP-induced PSA mRNA expression. However, the inhibition of ATF3 expression interrupted PSA suppression by HX109 (Figure 6D). 

Next, the effects of HX109 on CaMKKβ activity were evaluated by western blot, as CaMKKβ is known to inhibit AR-mediated gene expression and AR transcriptional activity [11]. TP had no effects on CaMKKβ phosphorylation in LNCaP cells, but treatment with HX109 increased levels of phosphorylated CaMKKβ in a dose-dependent manner (Figure 6E). When cells were pre-treated with the CaMKKβ inhibitor STO-609, HX109 did not affect PSA expression, showing the important role of CaMKKβ in the HX109-mediated inhibition of PSAs (Figure 6F). Taken together, our data suggested that the inhibition of androgen-mediated gene expression by HX109 might be dependent on ATF3 and CaMKKβ.

### 3.7. Calcium Increase by HX109 Plays A Crucial Role in the Regulation of AR Transactivation

**Figure 7:**
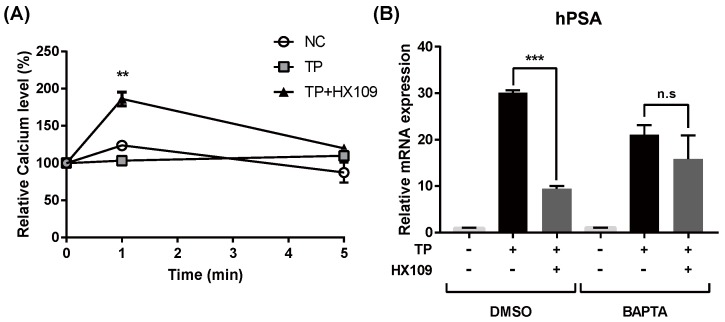
Calcium increase by HX109 plays a crucial role in the regulation of AR transcriptional activity. (**A**) Effects of HX109 on intracellular calcium levels. LNCaP cells were treated with 100 nM TP in the presence of HX109 1 mg/mL. Calcium levels were measured as described in the Materials and Methods section. ** *p* < 0.01 (one-way ANOVA) compared with TP only. (**B**) Effects of HX109 on calcium chelating condition. LNCaP cells were incubated with 20 μM BAPTA-AM for 30 min and treated with 100 nM TP in the presence of 1 mg/mL HX109 for 24 h. The RNA levels of hPSA were measured by quantitative RT-PCR. Values were normalized to GAPDH. *** *p* < 0.001 (one-way ANOVA) compared with TP only. n.s, not significant. All data are shown as mean ± S.E.M. of three independent experiments. ‘+’ means ‘treated with TP or HX109’, if it is on the right side of TP, it means ‘treated with TP’; if it is on the right side of HX109, it means ’treated with HX109’. ‘−’ means ‘not treated with TP or HX109’.

The binding of Ca^2+^/Calmodulin to CaMKKβ increases its enzymatic activity. To test whether HX109 regulates intracellular calcium levels, calcium assays were performed using cell lysates. When cells were treated with HX109, intracellular calcium levels increased at 1 min and decreased at 5 min (Figure 7A). The role of calcium in HX109’s effect on PSAs was confirmed in the Ca^2+^-free conditions. When cells were pretreated with the extracellular/intracellular calcium chelator BAPTA-AM, the HX109-mediated downregulation of PSA expression was inhibited (Figure 7B). However, pretreatment with EGTA, an extracellular specific calcium chelator, did not affect the effect of HX109 on PSA expression (see Figure S6 in the Supplementary Materials). These results indicated that HX109 might increase intracellular calcium to inhibit AR-mediated gene expression.

## 4. Discussion

In this study, we showed that the botanical formulation HX109 could ameliorate TP-induced prostate hyperplasia by controlling androgen receptor signaling. Oral administration of HX109 reduced the TP-induced increase in weight and PSA levels of the prostate. Furthermore, in LNCaP cells, HX109 inhibited androgen-induced proliferation and repressed androgen receptor-mediated gene expression. It appeared that these effects were mediated through an increase in the levels of ATF3 expression, phosphorylation of CaMKKβ, and also an increase in calcium levels, as the effects of HX109 were attenuated by treatment with ATF3-specific siRNA, CaMKKβ inhibitor, or calcium chelator. 

Androgen binds to AR, translocates to the nucleus, and binds to ARE present in the promoters of various genes, eventually leading to cell proliferation. Various molecules known to inhibit androgen signaling exert their effects by blocking AR-androgen interaction, AR degradation, or AR translocation. However, HX109 did not seem to use conventional pathways to exert its effect. Instead, HX109 appeared to repress AR transactivation by upregulating the AR-interacting factors ATF3 and CaMKKβ. This indirect regulation of AR signaling by HX109 might have advantages, as it might produce lesser side effects than finasteride, which directly modulates AR signaling by inhibiting DHT [30].

It has been reported that intracellular calcium levels are regulated by the influx of external calcium, by calcium channel openings, and by the release of calcium stored in endoplasmic reticulum (ER) [31]. In this study, pretreatment with EGTA, an extracellular calcium chelator, exerted no effect, while BAPTA-AM suppressed HX109-mediated PSA reduction. Therefore, the HX109-mediated increase in intracellular calcium appears to be the result of calcium release from ER, rather than influx from the outside. It has been reported that *Taraxacum officinale* can raise calcium levels through the regulation of ER [32]. Further studies are needed to clarify the exact mechanism underlying the HX109-mediated regulation of calcium levels in the cells.

Increases in intracellular calcium levels resulting from HX109 action may activate CaMKKβ by increasing its phosphorylation, as evidenced by our data. Since CaMKKβ is well known to repress AR-mediated gene expression [11], the activation of CaMKKβ may play a crucial role in the HX109-mediated suppression of androgen signaling. In addition, CaMKKβ regulates the phosphorylation of AMP-activated protein kinase (AMPK), which has been shown to inhibit prostate cell growth and AR activity [33,34]. Therefore, it is possible that HX109 may control AMPK through the activation of CaMKKβ, thereby producing the therapeutic effects observed in this study. 

ATF3 is expected to target BPH pathogenesis by mitigating oxidative stress and inflammation, or inhibiting androgen signaling [35]. Furthermore, the levels of ATF3 expression in the prostates of BPH patients have been shown to be lower than those of healthy control groups [29]. We showed that HX109 upregulated ATF3 expression, suppressing AR-mediated gene expression and eventually prostate enlargement. It would be interesting to investigate how HX109 would upregulate ATF3 expression at molecular levels.

We have not yet identified the compounds responsible for the observed effects of HX109 described in this study. The effects of HX109 might result from the complex actions of several components rather than the actions of one specific compound. For instance, flavonoids like quercetin and astragalin from *Cuscuta australis* have been shown to reduce oxidative stress in various cell types [36,37,38]. Notably, 7-hydroxydehydronuciferine and dauricine from *Nelumbo nucifera* have been reported to inhibit the proliferation of prostate cancer cells and urinary tract tumor cells [39,40]. Therefore, it is possible that the combined actions of various compounds contained in HX109 resulted in an inhibition of prostate enlargement. Given the significant effects of HX109, further studies are warranted to identify the active compounds, or at least a fraction with concentrated bioactivity, from this botanical extract.

Taken together, our data indicate that HX109 ameliorated TP-induced prostate enlargement and histological development. In the in vitro cell culture system, HX109 controlled AR-mediated gene expression and proliferation through the upregulation of ATF3, CaMKKβ, and intracellular calcium levels. The safety of the plants used for the preparation of HX109 has been established by a long history of human use. Indeed, no toxic effects of HX109 have been observed in acute or repeated-dose toxicity studies involving rats and dogs (unpublished data). Our data suggest that HX109 may have the potential to be a safe and efficacious therapeutic agent for BPH.

## Figures and Tables

**Table 1:** Effects of HX109 on body weight and prostate weight in testosterone propionate (TP)-induced BPH rats.

Group	Body Weight (g)		Prostate Weight (g)	Prostate Index (mg/100 g Body Weight)	Growth Inhibition (%)
	Initial	Final			
NC	356.93 ± 4.58	445.26 ± 5.42	0.0323 ± 0.0026	0.73 ± 0.063	
BPH	356.12 ± 5.79	436.29 ± 8.05	0.779 ± 0.025 ^####^	17.88 ± 0.64 ^####^	
HX109 200 mg/kg	356.56 ± 5.67	421.35 ± 10.15	0.686 ± 0.042	16.44 ± 1.28	8.4%
HX109 300 mg/kg	356.44 ± 5.09	432.98 ± 8.60	0.528 ± 0.036 ****	12.17 ± 0.72 ***	33.29%
Fina	536.05 ± 8.29	429.99 ± 11.26	0.454 ± 0.018 ****	10.55 ± 0.15 ****	42.74%

All data are mean ± standard error of the mean (S.E.M.). Growth inhibition = 100−((treated group−NC group)/(BPH group−NC group) × 100). NC (negative control): cottonseed oil subcutaneous injection(s.c.) + distilled water (DW) orally treated (p.o.); BPH: TP 3 mg/kg s.c. + DW p.o; HX109 200 mg/kg: TP s.c. + HX109 200 mg/kg p.o; HX109 300 mg/kg: TP s.c. + HX109 300 mg/kg p.o.; Fina: TP s.c. + Finasteride 10 mg/kg p.o.; ^####^ *p* < 0.0001 vs. NC group. *** *p* < 0.001, **** *p* < 0.0001 vs. BPH group.

## Supplementary Materials

The following are available online at http://www.mdpi.com/2072-6643/10/12/1946/s1, Figure S1: HPLC-ESI-Q-TOF-MS chromatogram of HX109 (negative ion mode); Figure S2: HPLC-ESI-Q-TOF-MS chromatogram of analytical sample for HX109 (positive ion mode); Figure S3: Cell-based bioassay of HX109 in LNCaP cells; Figure S4: Effects of HX109 on serum and prostate DHT level; Figure S5: Effects of HX109 on androgen target genes; Figure S6: Effects of HX109 on extracellular calcium chelating condition.
