# Supplementary material for: Botanical Formulation HX109 Ameliorates TP-Induced Benign Prostate Hyperplasia in Rat Model and Inhibits Androgen Receptor Signaling by Upregulating Ca2+/CaMKKβ and ATF3 in LNCaP Cells
Source: Nutrients. 2018 Dec 7;10(12):1946. doi: 10.3390/nu10121946 (PMC6316726; doi:10.3390/nu10121946)
Supplement: Supplementary file 1 [file nutrients-10-01946-s001.zip › Identification of plant species.pdf]

식물 종 동정 보고서

분석자	분석일시	보고서 작성자	확인 서명
김영기<br>김기중	2016.09.06~23	김기중	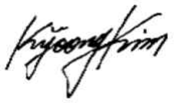

1. 접수일: 2016년 09월 06일 (소포접수)

2. 시료의 종 동정 의뢰자:
서울특별시 관악구 관악로 599번지 서울대학교 자연과학대학 기초과학연구동(203동) 5층 ㈜바이로메드 남인정

3. 분석의뢰내용: 시료 5종에 대한 종 동정의뢰

4. 분석장소: 고려대학교 생명과학대학 분자계통학연구실

*** 본 보고서는 의뢰한 5개 시료의 DNA 염기서열을 분석하여 그 결과를 기초로 서술된 보고서입니다. 속 및 근연종까지는 정확하게 동정되었으나, 명확한 종 동정을 위하여는 의뢰한 5개 시료 이외에 다수의 대조샘플들이 필요합니다. 따라서 본 자료는 법적 분쟁의 근거자료로 활용될 수 없음을 알려드립니다.**

1. 서론

의뢰자가 제공한 5개 식물 시료의 동정을 위하여 5개 시료의 형태적인 특징을 확인하고 DNA 분석을 수행하였다. 의뢰자로부터 제공받은 시료 중 1, 2번 샘플의 경우 건조된 잎, 화서 등이 포함된 혼합샘플이었고 3, 4, 5번 샘플은 종자였다. 모든 샘플은 정확한 DNA 추출을 위하여 실험용 알코올로 닦아낸 후에 추출에 사용하였다. 의뢰자로부터 시료는 2016년 9월 6일에 전달받았으며(그림 1), 추출하기 전 상태의 사진은 그림과 같다(그림 2,3,4,5,6).

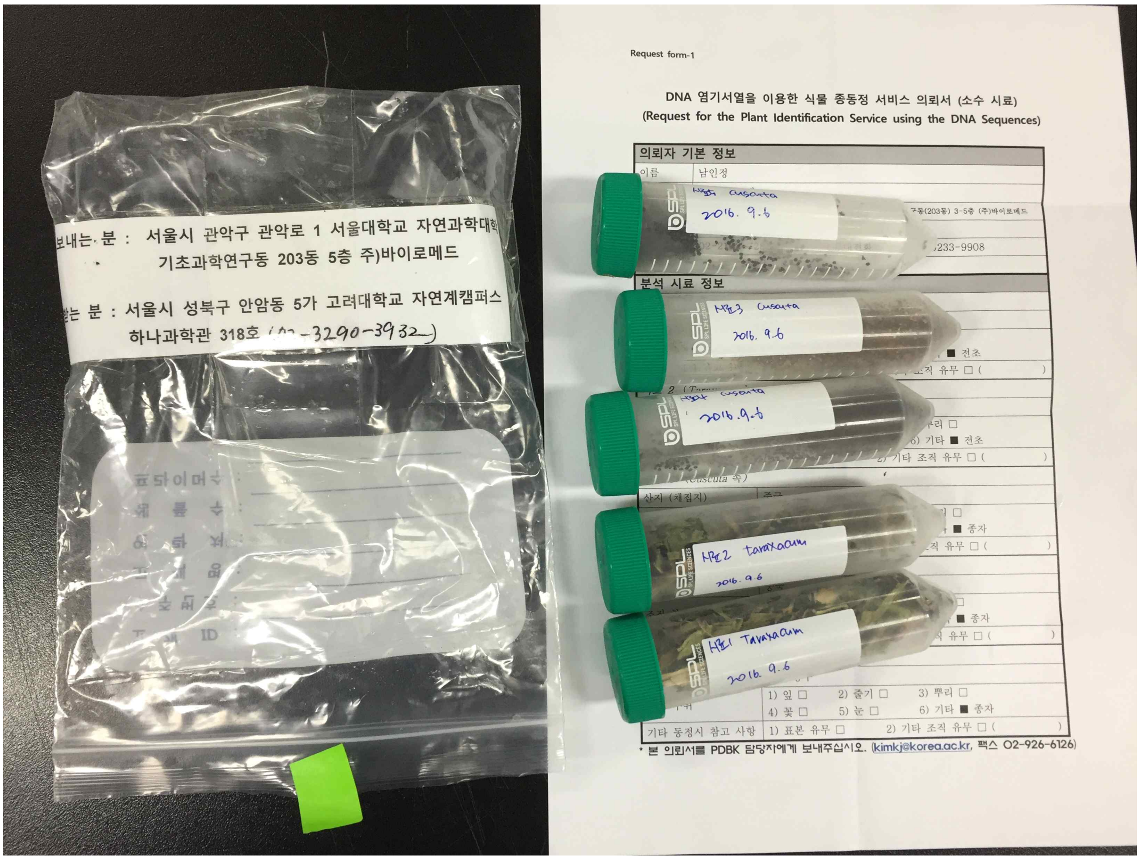

그림 1. 의뢰자로부터 수령한 5개 식물 시료

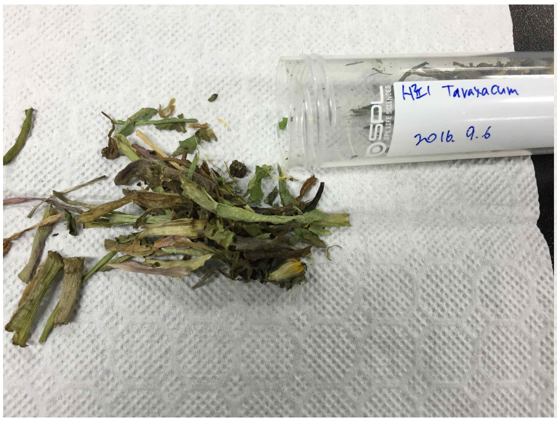

그림 2. 추출하기 전 시료 1

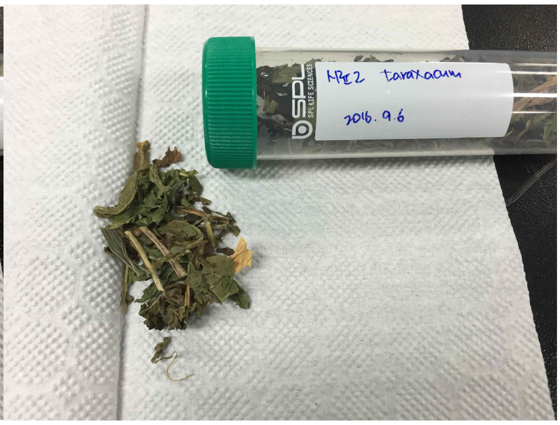

그림 3. 추출하기 전 시료 2

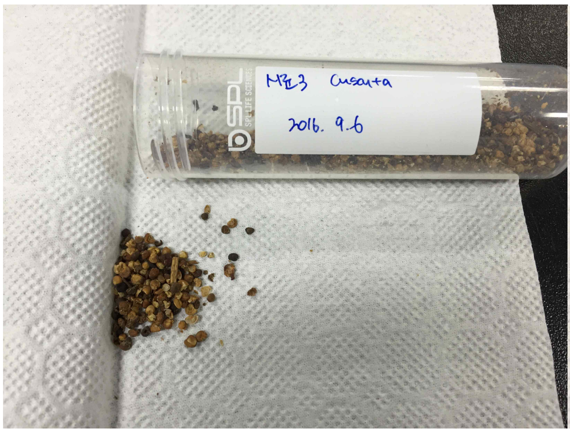

그림 4. 추출하기 전 시료 3

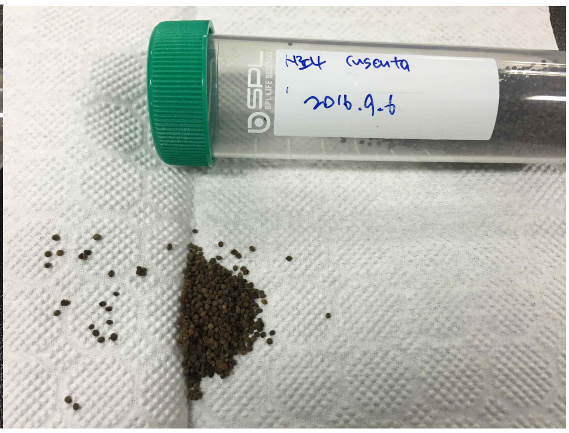

그림 5. 추출하기 전 시료 4

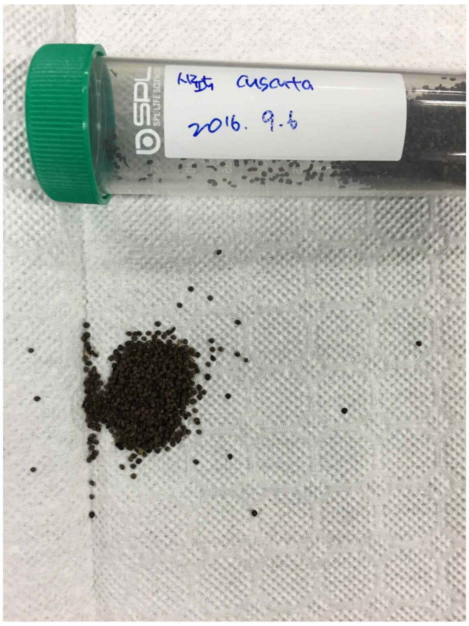

그림 6. 추출하기 전 시료 5

2. 재료 및 방법

1) DNA 추출 및 염기서열 결정

DNA는 식물의 잎과 줄기, 종자를 막자사발과 액체질소를 이용하여 분쇄하였다. 분쇄한 식물 시료는 DNeasy Plant Mini Kit(Qiagen, Germany)를 이용하여 추출하였다. DNA 염기서열은 식물 DNA Barcode 연구와 계통연구에 유용하게 사용되는 엽록체 유전체 3개 지역(*rbc*L, *trn*H-*psb*A, *psb*K-*psb*I)과 nrITS지역을 대상으로 PCR을 통해 증폭하고 그 염기서열을 결정하여 사용하였다. PCR 조건은 전 marker를 대상으로 동일하게 사용하였으며, 표 1에 정리하였다. 결정된 염기서열은 Geneious 6.1.8(Kearse et al., 2012)를 이용하여 조합하였다.

2) DNA 염기서열을 이용한 종 동정

정리된 염기서열은 Genbank(www.ncbi.nlm.gov/genbank)에 업로드된 염기서열과 Blast를

통하여 비교, 분석되었다. Genbank에 업로드된 새삼속, 민들레속 및 근연속들의 염기서열을 다운로드하여 시료와의 비교분석에 사용하였다. 결정한 염기서열 중 NCBI database에 충분하지않은 지역은 BLAST 결과값만을 분석에 이용하였다.

표 1. PCR 조건

Region	Step	Temperature	Time	Cycles
*rbc*L<br>*trn*H-*psb*A<br>*psb*K-*psb*I<br>ITS	Initial denaturation	94℃	3 min	1
	Denaturation	94℃	1 min	35
	Annealing	55℃	1 min	
	Extention	72℃	1 min	
	Elongation	72℃	7 min	1

3. 결과 및 고찰

1) *Taraxacum* 속 (시료 1, 2)

(1) 시료 1

PCR 결과 *rbc*L, *trn*H-*psb*A, *psb*K-*psb*I, ITS 4개 지역의 염기서열을 결정하였다. 결정된 염기서열을 BLAST를 이용하여 Genbank의 염기서열과 비교, 조사한 결과는 표 2와 같다. 표 2에서는 Maximum match score가 가장 높은 염기서열 3개만을 표시하였다.

표 2. 시료 1의 BLAST Report

GenBank BLAST Report			
*rbc*L(735 bp)	*trn*H-*psb*A(598 bp)	*psb*K-*psb*I(489 bp)	ITS(683 bp)
Taraxacum alpinum<br>1327 [i] 99% [ii]<br>KF602095.1 [iii]	*Taraxacum officinale*<br>972 [i] 99% [ii]<br>KU361241.1 [iii]	*Taraxacum officinale*<br>811 [i] 98% [ii]<br>KU361241.1 [iii]	*Taraxacum officinale*<br>1223 [i] 99% [ii]<br>AB766235.1 [iii]
Taraxacum officinale<br>1321 [i] 99% [ii]<br>KU361241.1 [iii]	*Taraxacum officinale*<br>946 [i] 99% [ii]<br>FJ395471.1 [iii]	*Lactuca sativa*<br>701 [i] 94% [ii]<br>DQ383816.1 [iii]	*Taraxacum mongolicum*<br>1216 [i] 99% [ii]<br>JN407433.1 [iii]
Taraxacum officinale<br>1321 [i] 99% [ii]<br>JX848432.1 [iii]	*Taraxacum officinale*<br>918 [i] 99% [ii]<br>HE966842.1 [iii]	*Lactuca sativa*<br>701 [i] 94% [ii]<br>AY865171.1 [iii]	*Taraxacum mongolicum*<br>1208 [i] 99% [ii]<br>AY548210.1 [iii]

i) Maximum match Scores ii) Maximum similarities iii) Accession Num.

Genbank에 등록된 염기서열과 시료 1로부터 얻은 염기서열을 비교 조사한 결과, *rbc*L 유전자에서는 *Taraxacum alpinum*이, *trn*H-*psb*A, *psb*K-*psb*I, ITS 지역에서는 *T. officinale*가 가장 높은 Maximum match Score를 기록하였다. *psb*K-*psb*I 지역에서 다른 속의 식물이 가장 유사도가 높게 나타난 것은 GenBank에 등록되어있는 *Taraxacum* 속 *psb*K-*psb*I 염기서열

이 한 개만 존재하기 때문이다.

(2) 시료 2

PCR 결과 *rbc*L, *trn*H-*psb*A, *psb*K-*psb*I, ITS 4개 지역의 염기서열을 결정하였다. 결정된 염기서열을 BLAST를 이용하여 Genbank의 염기서열과 비교, 조사한 결과는 표 3와 같다. 표 3에서는 Maximum match score가 가장 높은 염기서열 3개만을 표시하였다.

표 3. 시료 2의 BLAST Report

GenBank BLAST Report			
*rbc*L(735 bp)	*trn*H-*psb*A(377 bp)	*psb*K-*psb*I(473 bp)	ITS(739 bp)
Taraxacum alpinum<br>1321 [i] 97% [ii]<br>KF602095.1 [iii]	*Taraxacum platycarpum*<br>617 [i] 96% [ii]<br>KP214515.1 [iii]	*Taraxacum officinale*<br>784 [i] 98% [ii]<br>KU361241.1 [iii]	*Taraxacum officinale*<br>1315 [i] 98% [ii]<br>AB766235.1 [iii]
Taraxacum officinale<br>1315 [i] 97% [ii]<br>KU361241.1 [iii]	*Taraxacum officinale*<br>606 [i] 96% [ii]<br>FJ493264.1 [iii]	*Lactuca sativa*<br>673 [i] 93% [ii]<br>DQ383816.1 [iii]	*Taraxacum mongolicum*<br>1303 [i] 99% [ii]<br>AY548210.1 [iii]
Taraxacum officinale<br>1315 [i] 97% [ii]<br>JX848432.1 [iii]	*Taraxacum officinale*<br>582 [i] 95% [ii]<br>HE966842.1 [iii]	*Lactuca sativa*<br>673 [i] 93% [ii]<br>AY865171.1 [iii]	*Taraxacum mongolicum*<br>1301 [i] 99% [ii]<br>JN407433.1 [iii]

i) Maximum match Scores ii) Maximum similarities iii) Accession Num.

Genbank에 등록된 염기서열과 시료 2로부터 얻은 염기서열을 비교 조사한 결과, *rbc*L 유전자에서는 *T. alpinum*이, trnH-*psb*A 지역에서는 *T. platycarpum*이, *psb*K-*psb*I, ITS 지역에서는 *T. officinale*가 가장 높은 Maximum match Score를 기록하였다. *psb*K-*psb*I 지역에서 다른 속의 식물이 가장 유사도가 높게 나타난 것은 GenBank에 등록되어있는 *Taraxacum* 속 *psb*K-*psb*I 염기서열이 한 개만 존재하기 때문이다.

(3) 고찰

Genbank에 등록된 *Taraxacum* 속 염기서열과 *Taraxacum*이 속한 Cichorieae의 ITS 염기서열 512개를 내려받아 Maximum Likelihood Tree를 작성하는 데 이용하였다. 그 결과 시료 1은 *T. officinale, T. carpaticum, T. macrolepium, T. bulgaricum*과 단계통을 이루었다. 시료 2는 Tree에서 *Taraxacum* 속이 아닌 *Sonchus* 속과 단계통을 이루는 것을 확인하였다. 국내에서 *T. alpinum*은 보고된 바가 없다.

ML Tree의 결과를 확인하고 시료 2의 상태를 확인하였는데, 한 종의 잎이 아닌 여러 종의 잎이 섞여있는 것을 확인하였다. *Sonchus, Pilosella, Lapsana, Youngia, Hieracium* 속과 같은 식물들이 섞인 혼합시료로 판단된다.

이상의 결과를 종합하여 볼 때, 시료 1은 서양민들레(*Taraxacum officinale*)로 판단된다. 시료 2의 경우에는 4개 marker 모두에서 서양민들레(*T. officinale*)가 주요 부분을 차지하지만, 방가지똥, 고들빼기, 조밥나물 등의 혼합물이 섞여있는 혼합시료로 판단된다.

2) *Cuscuta* 속(시료 3, 4, 5)

PCR 결과 *trn*H-*psb*A, ITS의 2개 지역의 염기서열을 결정하였다. 결정된 염기서열을 BLAST를 이용하여 Genbank의 염기서열과 비교, 조사한 결과는 표 4, 5, 6과 같다. 표4, 5, 6에서는 Maximum match score가 가장 높은 염기서열 3개만을 표시하였다.

표 4. 시료 3의 BLAST Report

GenBank BLAST Report	
*trn*H-*psb*A(249 bp)	ITS(661 bp)
Cuscuta reflexa<br>289[i] 90%[ii] KP214515.1[iii]	*Cuscuta japonica*<br>1114[i] 96%[ii] DQ211588.1[iii]
Cuscuta reflexa<br>289[i] 90%[ii] X67512.1[iii]	*Cuscuta japonica*<br>1110[i] 98%[ii] KC542379.1[iii]
Cuscuta exaltata<br>237[i] 87%[ii] EU189132.1[iii]	*Cuscuta japonica*<br>1068[i] 98%[ii] DQ924571.1[iii]

표 5. 시료 4의 BLAST Report

GenBank BLAST Report	
*trn*H-*psb*A(267 bp)	ITS(660 bp)
Cuscuta obtusiflora<br>363[i] 95%[ii] EU189133.1[iii]	*Cuscuta australis*<br>1197[i] 99%[ii] KT383065.1[iii]
Cuscuta sandwichiana<br>363[i] 95%[ii] AY936347.1[iii]	*Cuscuta australis*<br>1195[i] 99%[ii] KT383068.1[iii]
Cuscuta gronovii<br>340[i] 94%[ii] AM711639.1[iii]	*Cuscuta australis*<br>1192[i] 99%[ii] KT383087.1[iii]

표 6. 시료 5의 BLAST Report

GenBank BLAST Report	
*trn*H-*psb*A(267 bp)	ITS(649 bp)
Cuscuta obtusiflora<br>363[i] 95%[ii] EU189133.1[iii]	*Cuscuta australis*<br>1194[i] 99%[ii] KT383068.1[iii]
Cuscuta sandwichiana<br>363[i] 95%[ii] AY936347.1[iii]	*Cuscuta australis*<br>1194[i] 99%[ii] KT383065.1[iii]
Cuscuta gronovii<br>340[i] 94%[ii] AM711639.1[iii]	*Cuscuta australis*<br>1188[i] 99%[ii] KT383087.1[iii]

(1) 고찰

Genbank에 등록된 염기서열과 비교분석한 BLAST 결과는 *trn*H-*psb*A지역에서는 *C. obtusiflora, C. sandwichiana, C. gronovii, C. reflexa*가 가장 유사한 것으로 나타났다. ITS 지역에서는 시료 3은 *C. japonica*가, 시료 4와 5는 *C. australis*가 가장 유사한 것으로 나타났

다.

Genbank에 등록된 *Cuscuta* 속 ITS 지역 염기서열 181개와 근연속 *Calystegia*의 염기서열 두 개를 내려받아 ML tree 분석에 이용하였다. 그 결과 시료 3은 *C. japonica*와 단계통을, 시료 4와 5는 *C. australis*와 단계통을 이루는 것을 확인하였다.

우리나라에 보고된 *Cuscuta*속 식물은 총 4종으로 *C. australis, C. japonica, C. chinensis, C. pentagona* 이다. 중국에는 보다 많은 11종이 기재되어 있는데(Flora of china, Convolvulaceae) *C. europaea, C. approximata, C. chinensis, C. australis, C. campestris, C. japonica, C. lupuliformis, C. macrolepis, C. monogyna, C. reflexa, C. gigantea* 가 그 학명이다. 우리나라와 중국에서 보고된 *Cuscuta*속 식물에 *trn*H-*psb*A지역 염기서열을 BLAST하여 얻어진 종은 포함되어 있지 않다.

이상의 결과를 종합하여 볼 때, 시료 3은 새삼(*C. japonica*), 시료 4와 5는 실새삼(*C. australis*)로 판단된다.
