# Supplementary material for: Botanical Formulation HX109 Ameliorates TP-Induced Benign Prostate Hyperplasia in Rat Model and Inhibits Androgen Receptor Signaling by Upregulating Ca2+/CaMKKβ and ATF3 in LNCaP Cells
Source: Nutrients. 2018 Dec 7;10(12):1946. doi: 10.3390/nu10121946 (PMC6316726; doi:10.3390/nu10121946)
Supplement: Supplementary file 1 [file nutrients-10-01946-s001.zip › S Lim (2018) supplement figure.pdf]

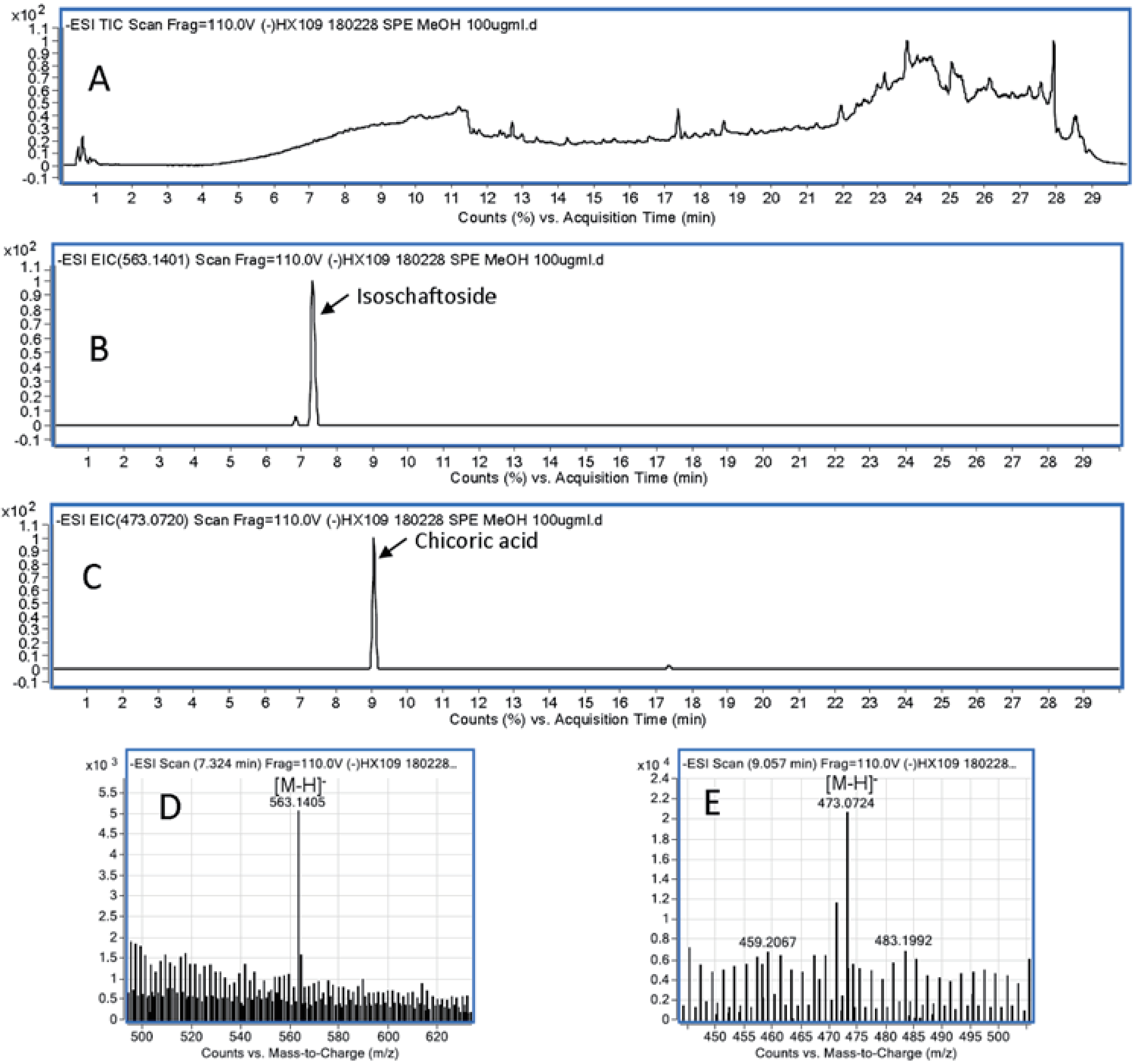

Supplement Figure 1. HPLC-ESI-Q-TOF-MS chromatogram of HX109 (negative ion mode).

The theoretical MS of isoshcaftoside and chicoric acid are 563.1401 $[M-H]^-$ and 473.0720 $[M-H]^-$, respectively. (A) Total ion chromatogram of HX109. (B) Extracted ion chromatogram of HX109 at *m/z* 563.1401. (C) Extracted ion chromatogram of HX109 sample at *m/z* 473.0720. (D) and (E) MS spectrum of mass peak at 7.324 and 9.057 min. The mass spectrum of both peaks showed $[M-H]^-$ ion peaks at *m/z* 563. 1405 and 473.0724, respectively. The error rate was within 5 ppm.

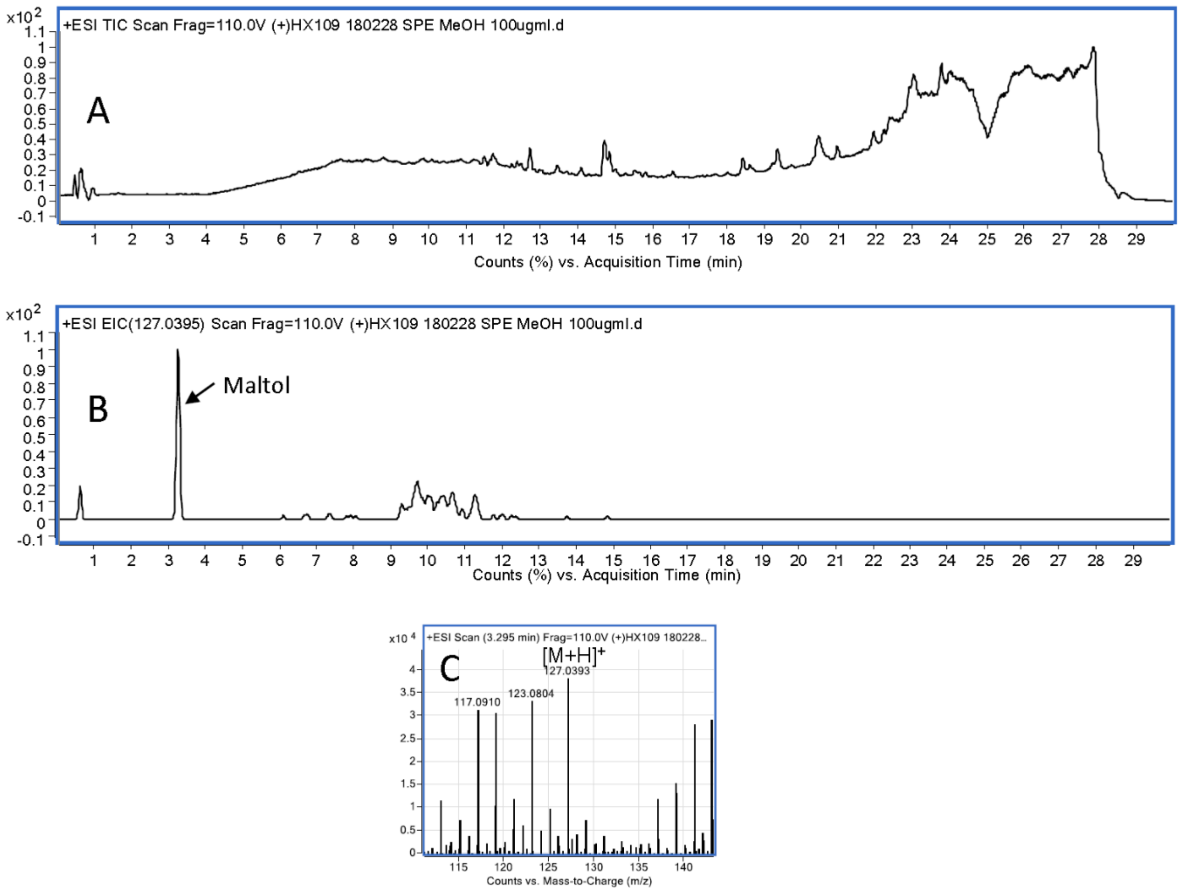

Supplement Figure 2. HPLC-ESI-Q-TOF-MS chromatogram of analytical sample for HX109 (positive ion mode).

The theoretical MS of maltol is *m/z* 127.0395 $[M+H]^+$. (A) Total ion chromatogram of HX109 sample; (B) Extracted ion chromatogram of HX109 sample at *m/z* 127.0395; (C) MS spectrum of mass peak at 3.295 min. The error rate was within 5ppm.

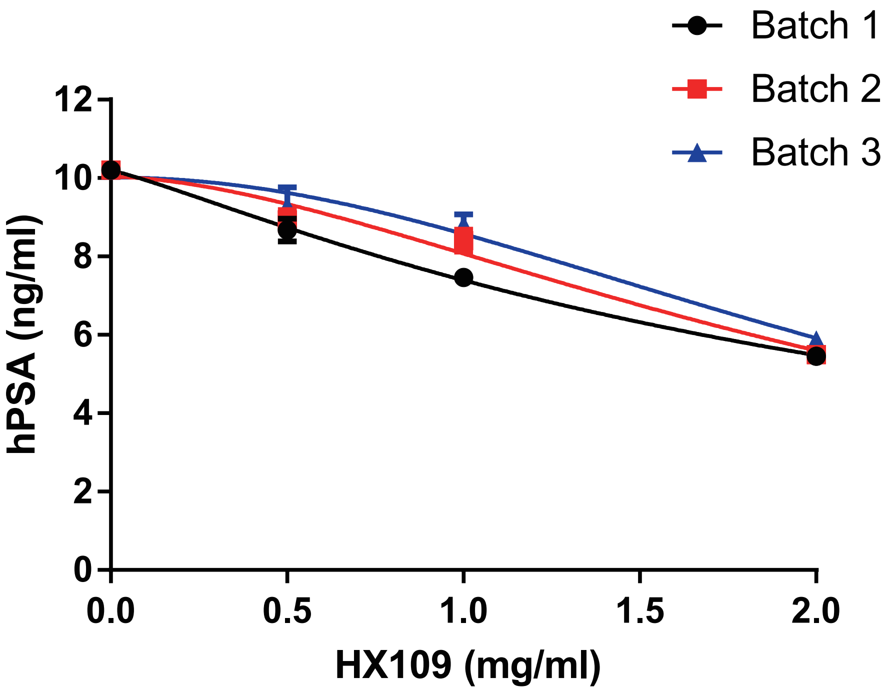

Batch	IC50 (mg/ml)
1	2.273
2	2.294
3	2.395

Supplement Figure 3. Cell-based bioassay of HX109 in LNCaP cells

LNCaP cells were treated as described in Fig. 4A. The effects of different batches of HX109 on hPSA protein secretion were analyzed by ELISA after 24 h. Half maximal inhibitory concentration (IC50) values of three batches were calculated from the graph. Data are shown as mean ± SEM of three independent experiments.

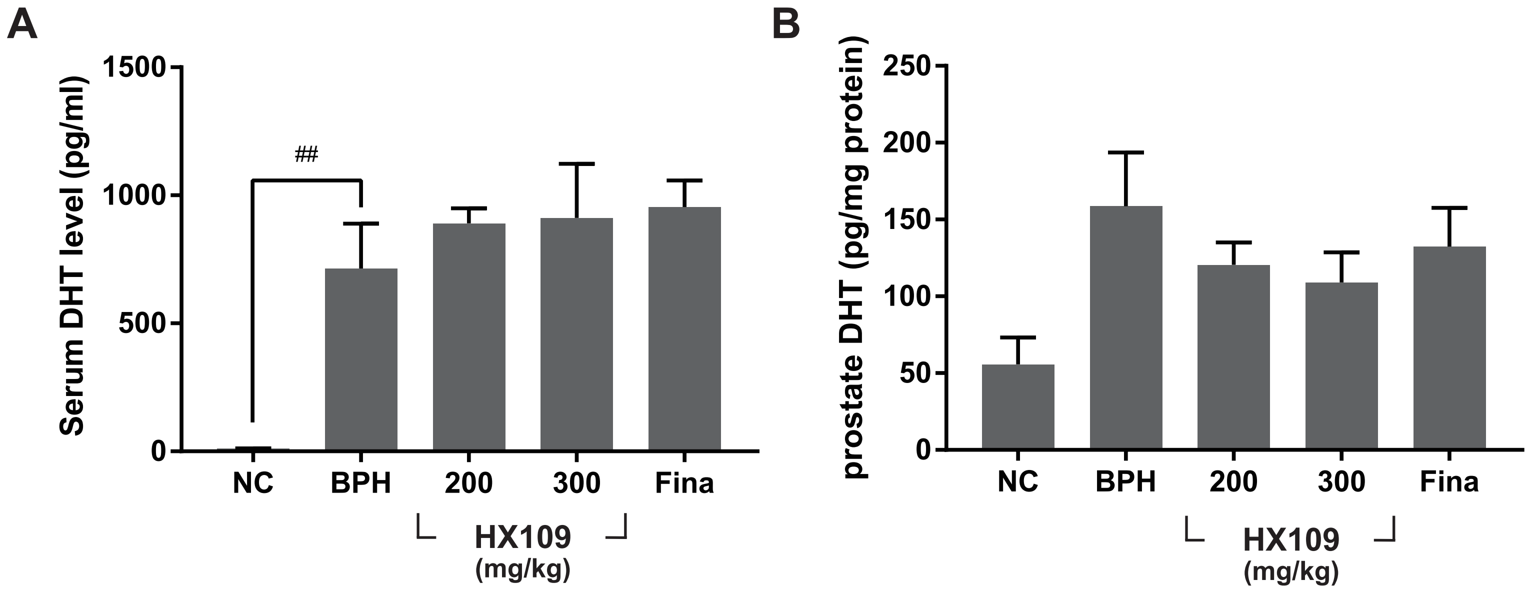

Supplement Figure 4. Effects of HX109 on serum and prostate DHT level

Castrated Sprague Dawley rats were injected with TP 3 mg/kg every three days and orally administrated with TDW (BPH) or HX109 200 mg/kg or HX109 300 mg/kg or Finasteride 5 mg/kg (Fina). Rats injected with vehicle were used as negative control (NC) group. After rats were sacrificed, the DHT levels of rat serum (A) or prostate (B) were measured by ELISA. ##$p<0.01$ (one-way ANOVA) compared with the NC group. n=5 per group. All Data are shown as mean ± SEM.

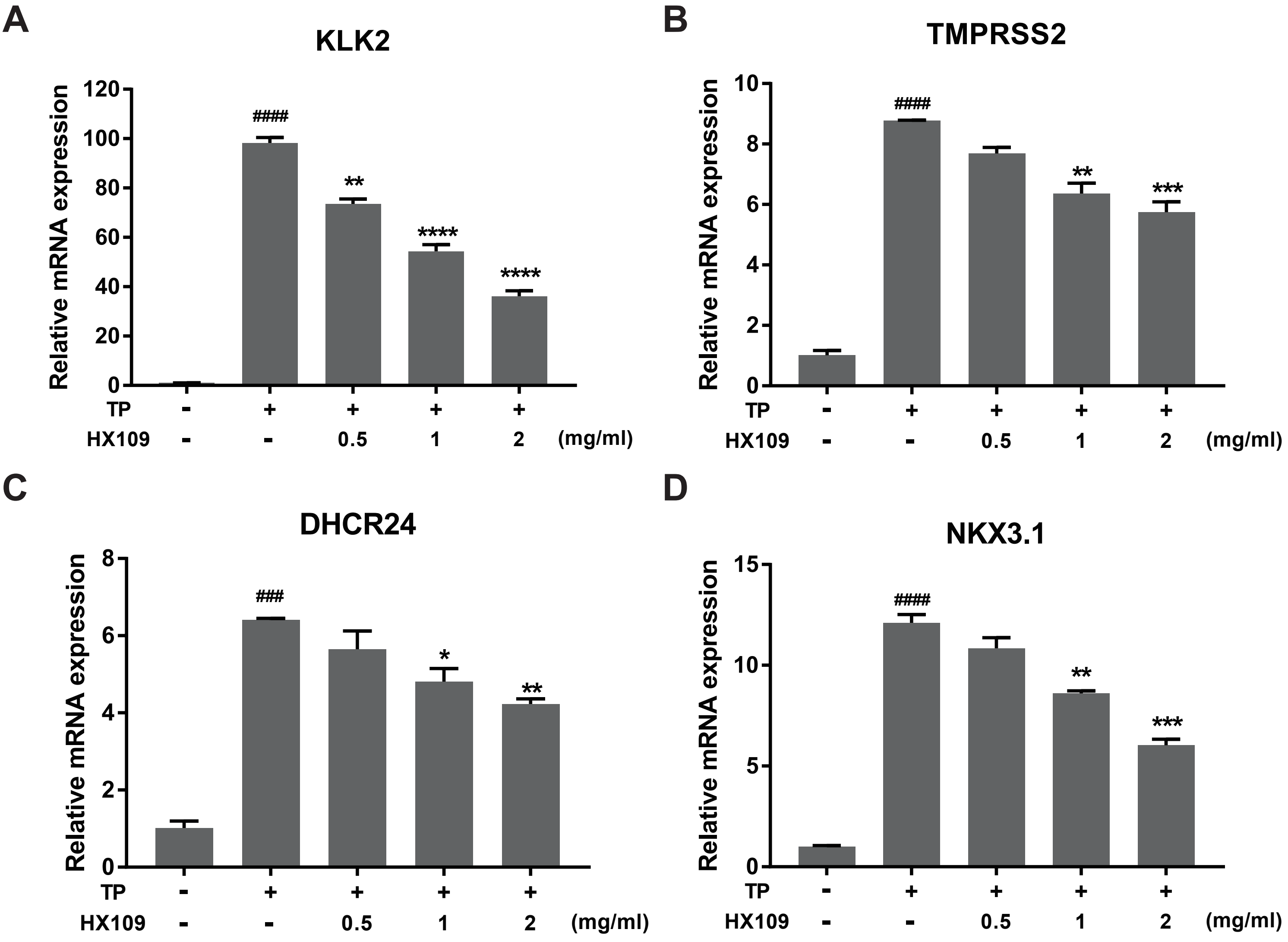

Supplement Figure 5. Effects of HX109 on androgen target genes

LNCaP cells were plated with culture media for 24 h, followed by androgen starvation by using phenol red free RPMI1640 containing 10% charcoal-stripped serum for 24h. After androgen starvation, LNCaP cells were treated with 100 nM TP and cultured in the presence of various concentrations of HX109 for 24 h. Total RNA was prepared, and RNA levels of KLK2 (A), TMPRSS2 (B), DHCR24 (C) and NKX3.1(D) were analyzed by quantitative RT-PCR. ####p<0.0001 compared with control, *p<0.05, ***p<0.001, ****p<0.0001 (one-way ANOVA) compared with TP only. All data are shown as mean ± SEM of three independent experiments.

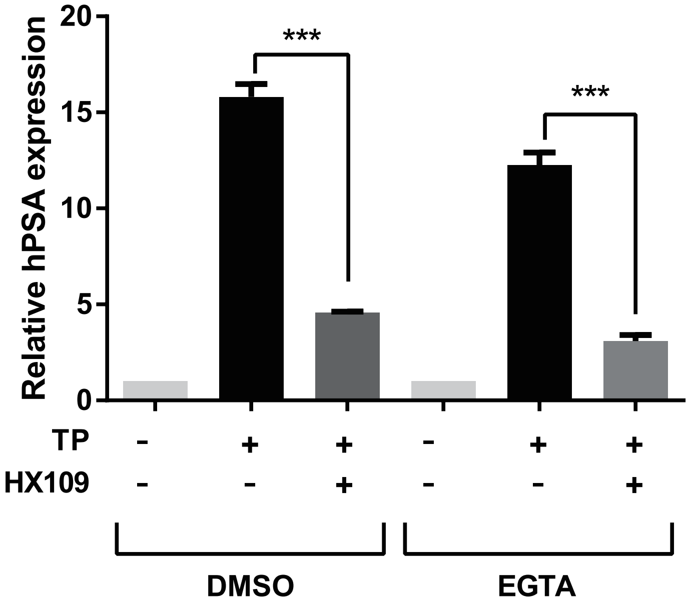

Supplement Figure 6. Effects of HX109 on extracellular calcium chelating condition.

LNCaP cells were incubated with 5 mM EGTA for 30 min and treated with 100 nM TP in the presence of HX109 1 mg/ml for 24 h. The RNA levels of hPSA were measured by quantitative RT-PCR. Values were normalized to GAPDH. ***p<0.001 (one-way ANOVA) compared with TP only. All Data are shown as mean ± SEM of three independent experiments.
